# **Reticular Dysgenesis in an Extremely Low Birth Weight Infant: A Case Report**

**DOI:** 10.1007/s10875-026-02029-7

**Published:** 2026-05-14

**Authors:** Kaoru Kinoshita, Hideki Muramatsu, Manabu Wakamatsu, Kazuto Ueda, Yoshiaki Sato, Yoshiyuki Takahashi

**Affiliations:** 1 Department of Pediatrics, Nakatsugawa Municipal General Hospital, Nakatsugawa, Japan; 2 Department of Pediatrics, Nagoya University Graduate School of Medicine, Nagoya, Japan; 3 Division of Neonatology, Center for Maternal-Neonatal Care, Nagoya University Hospital, Nagoya, Japan

## To the Editor

Reticular dysgenesis (RD) is a rare and fatal severe combined immunodeficiency (SCID) disorder caused by biallelic variants in *AK2*. It presents as profound neutropenia, severe T-cell lymphopenia, and frequent sensorineural hearing loss. Affected neonates usually die during early infancy unless they receive hematopoietic stem cell transplantation (HSCT) [1]. Newborn screening for SCID by quantification of T-cell receptor excision circles (TREC) has enabled earlier diagnosis, and some screening programs also incorporate quantification of kappa-deleting recombination excision circles (KREC) [2]. However, interpretation of these tests in preterm neonates is challenging because physiologically reduced lymphopoiesis often results in low TREC/KREC levels, which may lead to false positives [3]. Here, we report the survival of an extremely low birth weight infant (ELBWI) with genetically confirmed RD who was treated using a staged transplant strategy. To our knowledge, this is the first report of an ELBWI with RD detected by a combined TREC/KREC newborn screening program achieving successful immune reconstitution.

## Case Description

**Fig. 1:**
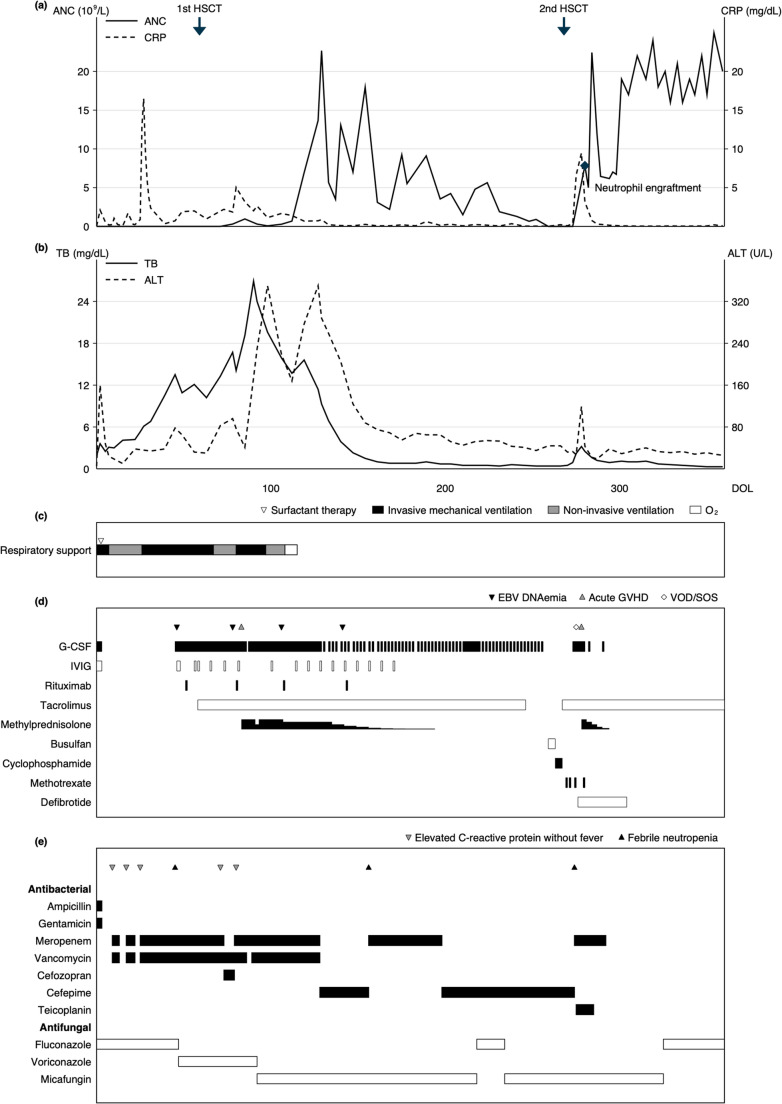
Clinical course from day of life (DOL) 0 to hospital discharge (DOL 359), (a) Absolute neutrophil count (ANC, solid line; left axis) and C-reactive protein (CRP, dashed line; right axis), with 1st and 2nd HSCT indicated by arrows. Neutrophil engraftment is marked at DOL 280. (b) Total bilirubin (TB, solid line; left axis) and alanine aminotransferase (ALT, dashed line; right axis). (c) Respiratory support over time (black: invasive mechanical ventilation; gray: non-invasive ventilation; white: oxygen), with blank periods indicating room air; surfactant therapy is shown by a downward triangle. (d) Transplant-related complications and immunomodulatory/conditioning therapies. Complication symbols: downward black triangle, EBV DNAemia; upward gray triangle, acute GVHD; white diamond, VOD/SOS. Horizontal bars indicate treatment periods (G-CSF, IVIG, rituximab, tacrolimus, methylprednisolone, busulfan, cyclophosphamide, methotrexate, and defibrotide); methylprednisolone dose started at 2 mg/kg/day and was subsequently tapered; bar height represents dose (mg/kg/day). (e) Anti-infective treatment timeline. Antibacterial and antifungal agents are shown as horizontal bars. Infection-related events are indicated at the top of the panel: downward gray triangle, elevated C-reactive protein without fever; upward black triangle, febrile neutropenia. Abbreviations: HSCT, hematopoietic stem cell transplantation; EBV, Epstein-Barr virus; GVHD, graft-versus-host disease; VOD/SOS, veno-occlusive disease/sinusoidal obstruction syndrome; G-CSF, granulocyte colony-stimulating factor; IVIG, intravenous immunoglobulin

A female infant was born prematurely at 29 weeks’ gestation by cesarean section due to a nonreassuring fetal status. Her birth weight was 824 g, and initial evaluation revealed severe neutropenia, anemia, and thrombocytopenia. Auditory brainstem response testing indicated bilateral sensorineural hearing loss. Newborn screening performed on day of life (DOL) 2 revealed that TREC and KREC levels were undetectable (0 copies/10⁵ cells and 0 copies/10⁵ cells, respectively), and this was confirmed by repeat testing on DOL 11 and 29. Flow cytometry analysis on DOL 8 showed a near-absence of circulating T-cells. Whole-exome sequencing identified compound heterozygous *AK2* exon 3 variants (c.308G > A [p.Arg103Gln] and c.307C >T [p.Arg103Trp]), confirming the diagnosis of RD. Therefore, the patient was managed under enhanced protective isolation in a private positive-pressure room. Enteral feeding was initially started with breast milk but was changed to a standard formula on DOL 22 because of concerns regarding cytomegalovirus transmission. In the early neonatal course, complications such as respiratory distress syndrome, with subsequent progression to chronic lung disease, as well as meconium ileus and cholestatic jaundice, developed (Fig. 1). Despite intensive supportive care, including G-CSF administration, severe neutropenia persisted (Fig. 1). On DOL 51, Epstein–Barr virus (EBV) DNAemia suggestive of primary EBV infection was detected, prompting rituximab administration.

Given her extreme prematurity and hepatic dysfunction, myeloablative conditioning was deemed infeasible. On DOL 59, she underwent an unrelated human leukocyte antigen (HLA)-matched cord blood transplantation without conditioning (Table 1). Although this resulted in donor-derived lymphoid reconstitution, durable myeloid engraftment failed, leading to persistent neutropenia and mixed chimerism. The patient also experienced complications, including EBV reactivation and acute graft-versus-host disease (GVHD), which were managed with rituximab, tacrolimus, and corticosteroids, respectively. Cholestasis was initially attributed to intestinal failure–associated liver disease resulting from extreme prematurity and prolonged parenteral nutrition. However, it worsened abruptly after transplantation, accompanied by the development of a skin rash, suggesting superimposed hepatic GVHD. Nonetheless, through GVHD-directed therapy and the use of fish oil–based lipid emulsion (Omegaven®), it gradually improved.

**Table 1:** Clinical characteristics and outcomes of the first and second cord blood transplantations

Parameter	1st HSCT	2nd HSCT
DOL of transplant	DOL 59	DOL 268
Height	36.5 cm	55.4 cm
Weight	1.67 kg	5.58 kg
Donor	Unrelated cord blood	Unrelated cord blood
HLA^a^	8/8 matched	7/8 matched
Cell dose		
TNC	58.3 × 10^7^/kg	16.9 × 10^7^/kg
CD34^+^ cells	27.0 × 10^5^/kg	8.80 × 10^5^/kg
Conditioning	None	BU + CY^b^
GVHD prophylaxis	Tacrolimus	Tacrolimus + sMTX
Neutrophil engraftment	DOL 119	DOL 280
Platelet engraftment	DOL 141	DOL 303
Acute GVHD	Grade IV (skin stage 3, liver stage 4)	Grade II (skin stage 3)
Chronic GVHD	None	None
VOD/SOS	None	Yes
Donor chimerism	DOL 252	DOL 439
Total PB MNC	61.7%	99.9%
PB CD3^+^ cells	97.4%	99.7%
PB CD14^+^ cells	45.7%	99.9%
PB CD19^+^ cells	NA	99.9%
PB CD56^+^ cells	NA	99.7%

^a^HLA matching was assessed at the allele level for HLA-A, -B, -Cw, and -DRB1

^b^BU: 3.48 mg per dose every 6 h for 4 days (PK-guided dosing; estimated cumulative AUC ≈ 63.0 mg·h/L based on test-dose pharmacokinetics and assuming linear pharmacokinetics); CY: 50 mg/kg/day for 4 days

Abbreviations: *HSCT* hematopoietic stem cell transplantation, *DOL* day of life, *HLA* human leukocyte antigen, *TNC* total nucleated cells, *BU* busulfan, *CY* cyclophosphamide, *GVHD* graft-versus-host disease, *sMTX* short-term methotrexate, *VOD/SOS* veno-occlusive disease/sinusoidal obstruction syndrome, *PB* peripheral blood, *MNC* mononuclear cells, *NA* not assessed

At 8 months old (DOL 268), during the same hospitalization following the first HSCT, the patient underwent busulfan and cyclophosphamide conditioning before receiving a second unrelated HLA-mismatched cord blood transplantation (Table 1). Engraftment was rapid, with neutrophil recovery on day 12 and complete donor chimerism confirmed at the day-14 evaluation. Early complications included veno-occlusive disease/sinusoidal obstruction syndrome, engraftment syndrome, and acute GVHD of skin, which responded to defibrotide and corticosteroid therapy. Several suspected infectious episodes responded to empiric antimicrobial therapy (Fig. 1); however, blood cultures remained negative, with no microbiologically documented invasive bacterial or fungal infections identified.

At 4 months after the second HSCT, the patient remained clinically stable and infection-free. She demonstrated robust multilineage engraftment with adequate immune reconstitution and no longer required immunoglobulin replacement. At the last follow-up at 19 months of age (corrected for gestational age, 16 months), the patient’s height was 74.2 cm (− 2.20 SD) and weight was 8.6 kg (− 1.52 SD), with no evidence of neurodevelopmental delay; additionally, a hearing aid had been applied to manage sensorineural hearing loss.

## Discussion

This case highlights several important points. First, TREC levels are often low in preterm neonates due to physiological immaturity, increasing the likelihood of false-positive results. In such cases, screening programs adopt a stepwise approach, whereby immunologic evaluation is deferred, and TREC testing is repeated during hospitalization. A structured protocol from Israel involved retesting every 2 weeks and only referring those with persistently low TREC levels for evaluation at discharge. However, because four of 16 such infants died before confirmatory testing, the possibility of undiagnosed immunodeficiency could not be excluded [4]. These findings underscore the risk of delaying evaluation in ELBWI with low TREC values solely based on gestational age. In our case, recognition of persistently undetectable levels of TREC and KREC led to prompt immunologic assessment and whole-exome sequencing, resulting in a definitive diagnosis of RD during the neonatal period and enabling timely therapeutic decision-making.

Second, our experience underscores the therapeutic dilemma of HSCT in ELBWI with RD. An international retrospective analysis of 32 patients with RD found that HSCT without myeloablative conditioning was uniformly unsuccessful in achieving durable myeloid engraftment, and the inclusion of a myeloablative agent, typically busulfan, was essential for long-term survival [5]. In our case, myeloablative conditioning was initially contraindicated due to organ immaturity and hepatic dysfunction. Thus, the first HSCT was performed without conditioning and was unable to achieve durable myeloid engraftment. However, donor-derived lymphoid reconstitution was achieved, offering protection against infections during a critical high-risk period. This bridging approach enabled patient survival until she could tolerate a busulfan-based regimen (at 8 months old), ultimately achieving complete multilineage engraftment and immune recovery.

Our case illustrates that combined TREC/KREC newborn screening enables early diagnosis of RD, even in extremely premature infants. Moreover, the successful outcome suggests that a staged transplant strategy of initial conditioning-free HSCT to establish lymphoid protection, followed by delayed myeloablative HSCT when feasible, is a life-saving approach in this otherwise fatal condition. Further data collection and collaborative research are necessary for refining treatment strategies and clarifying long-term outcomes.

## Data Availability

No datasets were generated or analyzed during the current study.

## Declarations

Written informed consent for publication of the clinical details was obtained from the patient’s parent/guardian. A copy of the consent form is available for review by the Editor of this journal.
